# Valve embolization during transcatheter aortic valve implantation: Incidence, risk factors and follow-up by computed tomography

**DOI:** 10.3389/fcvm.2022.928740

**Published:** 2022-07-22

**Authors:** David Frumkin, Malte Pietron, Andreas Kind, Anna Brand, Fabian Knebel, Michael Laule, David M. Leistner, Ulf Landmesser, Florian Krackhardt, Mohammad Sherif, Simon H. Sündermann, Herko Grubitzsch, Alexander Lembcke, Stefan M. Niehues, Karl Stangl, Henryk Dreger

**Affiliations:** ^1^Medizinische Klinik mit Schwerpunkt Kardiologie und Angiologie, Charité – Universitätsmedizin Berlin, Berlin, Germany; ^2^German Centre for Cardiovascular Research (DZHK), Partner Site Berlin, Berlin, Germany; ^3^Medizinische Klinik für Kardiologie, Charité – Universitätsmedizin Berlin, Berlin, Germany; ^4^Sana Klinikum Lichtenberg, Berlin, Germany; ^5^Medizinische Klinik mit Schwerpunkt Kardiologie, Charité – Universitätsmedizin Berlin, Berlin, Germany; ^6^Klinik für kardiovaskuläre Chirurgie, Charité – Universitätsmedizin Berlin, Berlin, Germany; ^7^Department of Cardiothoracic and Vascular Surgery, German Heart Center Berlin, Berlin, Germany; ^8^Klinik für Radiologie, Charité – Universitätsmedizin Berlin, Berlin, Germany

**Keywords:** transcatheter aortic valve replacement, complications, valve embolization, valve migration, valve dislocation

## Abstract

**Background:**

In most cases of transcatheter valve embolization and migration (TVEM), the embolized valve remains in the aorta after implantation of a second valve into the aortic root. There is little data on potential late complications such as valve thrombosis or aortic wall alterations by embolized valves.

**Aims:**

The aim of this study was to analyze the incidence of TVEM in a large cohort of patients undergoing transcatheter aortic valve implantation (TAVI) and to examine embolized valves by computed tomography (CT) late after TAVI.

**Methods:**

The patient database of our center was screened for cases of TVEM between July 2009 and July 2021. To identify risk factors, TVEM cases were compared to a cohort of 200 consecutive TAVI cases. Out of 35 surviving TVEM patients, ten patients underwent follow-up by echocardiography and CT.

**Results:**

54 TVEM occurred in 3757 TAVI procedures, 46 cases were managed percutaneously. Horizontal aorta (odds ratio [OR] 7.51, 95% confidence interval [CI] 3.4–16.6, *p* < 0.001), implantation of a self-expanding valve (OR 4.63, 95% CI 2.2–9.7, *p* < 0.01) and a left ventricular ejection fraction < 40% (OR 2.94, 95% CI 1.1–7.3, *p* = 0.016) were identified as risk factors for TVEM. CT scans were performed on average 26.3 months after TAVI (range 2–84 months) and detected hypoattenuated leaflet thickening (HALT) in two patients as well as parts of the stent frame protruding into the aortic wall in three patients.

**Conclusion:**

TVEM represents a rare complication of TAVI. Follow up-CT detected no pathological findings requiring intervention.

## Introduction

Transcatheter valve embolization and migration (TVEM) are potential complications of transcatheter aortic valve implantation (TAVI) (1). Recent data from two large retrospective cohorts suggest that TVEM is rare but associated with significantly increased morbidity and mortality (2, 3). Compared to a propensity-matched control cohort of TAVI patients, TVEM resulted in a significant increase of strokes at 30 days and a non-significant trend for a higher stroke rate 1 year after TAVI (2). Subclinical leaflet thrombosis is a frequent finding after TAVI and has been discussed as possible cause of embolic cardiovascular events (4, 5). Accordingly, leaflet thrombosis of embolized valves left in the ascending aorta might be of clinical significance. However, long-term imaging data on the fate of embolized valves is scarce. Therefore, the aim of our study was (i) to analyze the incidence, mechanisms and management of TVEM; (ii) to investigate the risk factors for TVEM and (iii) to examine embolized valves by electrocardiogram (ECG)-gated computed tomography (CT) for late complications such as leaflet and stent thrombosis or aortic wall alterations in a large cohort of TAVI patients.

## Materials and methods

### Study design

In this retrospective single-center cohort, we screened for cases of TVEM in 3757 consecutive patients undergoing TAVI from July 2009 to July 2021. TVEM was defined according to Valve Academic Research Consortium-2 (VARC-2) criteria (6), with valve embolization or migration during or after implantation taken as inclusion criteria.

Causes of TVEM were analyzed by review of procedural records and angiographic images. Cases with implantation of a second valve for the treatment of paravalvular leakage (PVL) or after deliberate removal of the first valve due to acute coronary obstruction were excluded from this study. The prevalence of potential risk factors for TVEM (including comorbidities and aortic root morphology) in our TVEM patients was compared to a control cohort of 200 consecutive patients who underwent TAVI from the period 12/2019 to 05/2020. To ensure that the sample cohort is representative of the total cohort, age and sex were compared. Surviving patients from the TVEM group were asked to participate in the imaging sub-study. Patients who gave consent were examined by transthoracic echocardiography (TTE) and CT to analyze morphologic features of the embolized valve and the surrounding aorta as well as the function of the secondary valve in aortic position.

The study was conducted in accordance with the Declaration of Helsinki and approved by the local ethics committee and the German Federal Office for Radiation Protection (registration number EA4/177/18).

### Angulation of the ascending aorta

Datasets of the planning CT performed prior to TAVI were analyzed using 3mensio Structural Heart 10.2 (Pie Medical Imaging BV, Maastricht, Netherlands). The angle between the ascending aorta and the aortic annulus plane was measured from a coronal projection at the level of the aortic annulus. Horizontal aorta was defined if angulation was > 48° as proposed previously (7).

### Echocardiography

A comprehensive transthoracic echocardiographic assessment was performed in accordance with the recommendations of the European Association of Cardiovascular Imaging (EACVI) for echocardiographic assessment of valve stenosis (8) and follow-up management after transcatheter aortic valve implantation (9). All echocardiographic studies were performed by experienced cardiologists on a Vivid E95 (GE Vingmed, Horten, Norway) system with a M5S 1.5–4.5 MHz transducer.

### Computed tomography

All follow-up examinations were performed using a 320-row-detector CT system (Aquilion ONE Vision, Canon Medical Systems, Otawara, Japan) and a two-step imaging protocol: a volume CT scan of the heart (“cardiac CT scan”) followed by a spiral CT scan of the thoracoabdominal arteries (“angio CT scan”). The cardiac CT scan was performed as ECG-gated data acquisition covering a full cardiac cycle (i.e., 0–99% of the RR-interval). The detector width was set at 16 cm to cover the entire heart and the ascending aorta in the craniocaudal direction.

All scanning was performed during inspiratory breath-hold at 135 kV tube voltage, 660 mA tube current, and at a gantry rotation time of 275 ms. All images were reconstructed using a standard soft tissue convolution kernel (FC 05) and the implemented iterative reconstruction algorithm (AIDR 3D, strong) at a slice thickness of 0.5 mm, an interval of 0.5 mm and an image matrix of 512 × 512. A total of 80 mL of contrast medium with an iodine content of 370 mg/mL (Ultravist 370; Bayer) was injected intravenously using a dual-head power injector (Dual Shot GX, Nemoto Kyorindo) at a flow rate of 4 ml/s followed by a saline chaser bolus of 40 ml injected with the same flow rate. The CT scans were then initiated using the scanners bolus tracking feature after reaching an attenuation of 200 HU in the descending aorta.

### Statistical analysis

Statistical Package for Social Studies (SPSS, IBM Corp, Released 2020, IBM SPSS Statistics for Mac OS, Version 27.0. Armonk, NY) was used for statistical analysis. Data were expressed as mean ± standard deviation for continuous variables or as percentage for categorical variables. The significance of differences in clinical data was calculated using the non-parametric Mann-Whitney U test for categorical variables and the Kolmogorov-Smirnov test for continuous variables. Absolute and relative incidence of TVEM in the overall TAVI population was determined. Logistic regression analysis was performed to assess associations of clinical data, type of transcatheter heart valves (THV) and imaging parameters with the occurrence of TVEM. Nagelkerkes R square was obtained to prove validation of the regression model. A *p*-value < 0.05 was considered statistically significant.

## Results

### Study population and clinical characteristics

**FIGURE 1:**
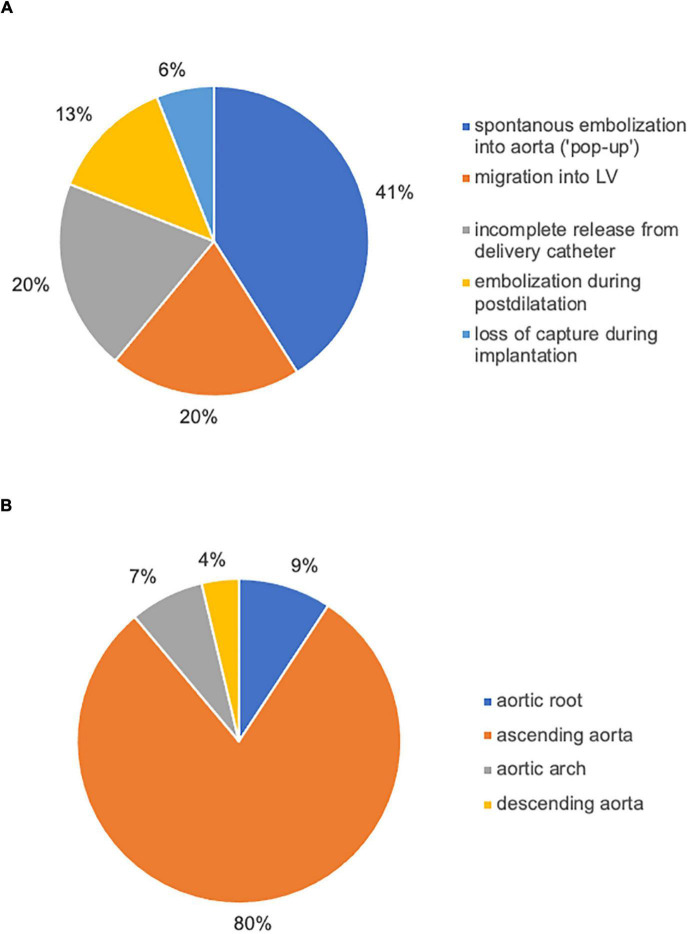
Causes of TVEM and the final position of embolized valves left *in situ*. Review of procedural records and angiograms identified five main mechanisms of TVEM during TAVI **(A)**. In the majority of cases, the embolized THV was left in the ascending aorta **(B)**.

Between July 2009 and July 2021, 3757 TAVI procedures were performed in our center. A total of 54 patients met VARC-2 Criteria for TVEM (TVEM group) corresponding to an overall incidence of TVEM of 1.44%. Incidence of TVEM in our registry was clustered in the beginning and then decreased rapidly over the years (up to 3.4% 2009–2013, 0.55% in 2020). 85.2% of TVEM occurred after implantation of a self-expanding valve (SEV), 14.8% after implantation of a balloon-expandable valve (BEV). Overall, the incidence of TVEM was significantly more frequent after implantation of a self-expanding valve compared to implantation of a balloon-expandable valve (2.3 vs. 0.4%, *p* < 0.001). The incidence of TVEM after SEV implantation did not change significantly after introduction of next-generation devices (*p* = 0.294, Corevalve vs. Evolut R/PRO and Portico). The valve types implanted during the study period and their respective incidence of TVEM are given in Table 1. 46 TVEM patients (85.2%) were treated by transcatheter implantation of a second valve while eight patients (14.8%) underwent conversion to surgery. Review of the procedural records and images revealed five major mechanisms for TVEM: (1) spontaneous embolization into the ascending aorta (“pop-up”), (2) migration into the LV, (3) accidental pull-back of the THV into the ascending aorta during removal of the delivery system due to incomplete release of the valve, (4) embolization during postdilatation, and (5) embolization due to loss of capture during implantation. The distribution of the mechanisms of TVEM and the final position of the embolized THV are provided in Figure 1.

**TABLE 1:** Distribution of valve types and incidence of transcatheter valve embolization and migration (TVEM).

Valve type	All patients	TVEM	Incidence of TVEM (%)
All	3757	54	1.44
Edwards Sapien XT	302	6	1.99
Edwards Sapien 3	1444	2	0.14
Medtronic Corevalve	625	17	2.72
Medtronic Evolut R/PRO	496	13	2.62
Abbott Portico/Navitor	806	16	1.99
other (Acurate Neo, Allegra, Directflow, Centera, Lotus)	84	0	0

To identify risk factors for TVEM, we compared clinical as well as anatomical and procedural characteristics of patients with TVEM with a cohort of 200 consecutive patients from the period 12/2019 to 05/2020 undergoing TAVI for native aortic valve disease. Comparison of age and sex between the sample cohort (age 80.4 years ± 6,3; 44.6% female sex) and the total study cohort (age 84.9 years ± 8.3; 51.9% female sex) showed good matching.

Clinical data of both cohorts are outlined in Table 2. Compared to the control cohort, significantly more TVEM patients had a history of heart failure with reduced ejection fraction (HFrEF). Age, sex, body mass index (BMI) and other comorbidities which might influence implantation techniques (e.g., renal failure) did not differ significantly between both groups. Anatomical factors like horizontal aorta, severe aortic regurgitation, as well as the use of a self-expanding valve showed significant differences between the groups and were further evaluated by regression analysis (Table 3).

**TABLE 2:** Baseline characteristics of all transcatheter valve embolization and migration (TVEM) patients and the control cohort.

Baseline characteristics	TVEM (*n* = 54)	Control cohort (*n* = 200)	*P*-value
Age, years	78.8 ± 10.5	80.6 ± 6.3	0.835
Female sex, *n* (%)	30 (55.6%)	92 (46.0%)	0.988
Body mass index, kg/m^2^	26.2 ± 4.8	27.6 ± 5.8	0.112
HFrEF (LVEF < 40%), *n* (%)	9 (16.7%)	13 (6.5%)	0.016
Arterial hypertension, *n* (%)	49 (90.7%)	192 (96.0%)	0.975
Prior permanent pacemaker implantation, *n* (%)	8 (14.8%)	26 (13.0%)	0.617
Prior stroke, *n* (%)	4 (7.4%)	17 (8.5%)	0.881
Chronic kidney disease, *n* (%)	22 (40.7%)	79 (39.5%)	0.638

HFrEF, heart failure with reduced ejection fraction; LVEF, left ventricular ejection fraction; TVEM, transcatheter valve embolization and migration.

**TABLE 3:** Anatomical characteristics and procedural data in patients with transcatheter valve embolization and migration (TVEM) compared to the control group.

Anatomical and procedural characteristics	TVEM (*n* = 54)	Control group (*n* = 200)	*P*-value
Annular diameter, mm	22.9 ± 1.5	24.3 ± 2.4	0.187
Severe aortic regurgitation, *n* (%)	2 (3.8%)	0 (0%)	0.005
Aortic valve area, cm^2^	0.73 ± 0.32	0.79 ± 0.33	0.067
Mean pressure gradient, mmHg	41.4 ± 18.1	41.6 ± 13.2	0.644
*V*_*max*_, m/s	4.00 ± 0.9	4.03 ± 0.65	0.646
Horizontal aorta, *n* (%)	35 (60.5%)	32 (16.0%)	<0.001
Valve type, *n* (%)			
Self-expanding	46 (85.2%)	105 (52.5%)	<0.001
Balloon-expandable	8 (14.8%)	95 (47.5%)	<0.001

V_max_, maximal velocity; TVEM, transcatheter valve embolization and migration.

Distribution of THV types (SEV vs. BEV) were similar in the overall cohort of 3757 TAVI patients and the control group of 200 patients: 2011 SEV (46.5%) and 1746 BEV (53.5%) in the total cohort vs. 105 SEV (52.5%) and 95 BEV (47.5%) in the control cohort.

### Risk factors for transcatheter valve embolization and migration

In a logistic regression analysis, age, sex, BMI, arterial hypertension, prior permanent pacemaker introduction, prior stroke and chronic kidney disease showed no significant relationship for the occurrence of TVEM (Table 4). Nagelkerke’s R square for the model was 0.497, showing good validation for the model. In contrast, the use of a SEV (*p* < 0.001, OR 4.63, 95% CI 2.2–9.7), the presence of a horizontal aorta (*p* < 0.001, OR 7.51, 95% CI 3.4–16.6) and HFrEF (*p* = 0.016, OR 2.94, 95% CI 1.1–7.3) were significantly associated with a higher risk for TVEM in the regression analysis (Table 4).

**TABLE 4:** Regression analysis of risk factors for transcatheter valve embolization and migration.

Baseline parameters	Odds ratio	95% CI	*P*-value
Female sex	1.62	0.45–5.73	0.469
Age (per year)	1.04	0.94–1.16	0.44
Body mass index (per kg/m^2^)	0.96	0.86–14.3	0.511
Arterial hypertension	1.11	0.82–13.77	0.937
Chronic kidney disease	2.07	0.63–6.80	0.231
Prior permanent pacemaker implantation	1.25	0.23–6.99	0.769
Prior stroke	0.73	0.07–7.33	0.788
HFrEF (LVEF < 40%)	2.94	1.10–7.30	0.016
Severe Aortic regurgitation	1.74	0.74–4.32	0.23
Aortic valve area (per mm^2^)	0.421	0.02–12.09	0.614
Mean pressure gradient (per mmHg)	1.01	0.96–1.05	0.77
Aortic annulus size (per mm^2^)	0.99	0.99–1.00	0.245
Use of self-expanding valve	4.63	2.21–9.73	<0.001
Horizontal aorta	7.51	3.41–16.55	<0.001

HFrEF, Heart failure with reduced ejection fraction; LVEF, left ventricular ejection fraction; TVEM, transcatheter valve embolization and migration; CI, confidence interval.

### Follow-up imaging

Out of 54 TVEM patients, none died during the index procedure, in-hospital mortality was 7.4% (4 patients). We tried to contact all patients which were identified as still alive and managed to get in contact with 22 patients. Out of these 22 patients, ten patients gave consent to undergo follow-up imaging by TTE and CT. Table 5 provides detailed information on the patients included in the imaging sub-study. In most cases, the embolized valves could not be sufficiently visualized by transthoracic echocardiography (TTE). TTE revealed normal function of all secondary THV in the aortic root.

**TABLE 5:** Overview of all patients examined by computed tomography (CT).

Patient	Age at TAVI (years)	Sex	Embolized THV	Mechanism of TVEM	Second THV	Follow-up (months)	Final position of THV	CT finding	Oral anticoagulation
1	73	m	Sapien XT 26 mm	Dislocation into aortic root after loss of capture during postdilation due to severe regurgitation	Sapien XT 29 mm	84	Aortic root	No pathological finding	Yes
2	78	m	CoreValve 29 mm	Valve pulled into ascending aorta due to incomplete release from delivery catheter	CoreValve 29 mm	57	Ascending aorta	No pathological finding	Yes
3	77	f	Portico 29 mm	Valve pulled into ascending aorta due to incomplete release from delivery catheter	Sapien 3 26 mm	43	Ascending aorta	Upper crown protruding into the aortic wall	No
4	79	f	Evolut R 26 mm	“Pop-up” after valve release	Evolut R 26 mm	37	Ascending aorta	Upper crown protruding into the aortic wall	No
5	76	f	Evolut R 26 mm	Valve pulled into ascending aorta due to incomplete release from delivery catheter	Sapien 3 23 mm	19	Ascending aorta	No pathological finding	Yes
6	84	f	Portico 27 mm	“Pop-up” after valve release	Sapien 3 23 mm	9	Ascending aorta	Upper crown protruding into the aortic wall	Yes
7	85	m	Sapien 3 Ultra 26 mm	Loss of capture during implantation	Sapien 3 Ultra 26 mm	6	Aortic arch	No pathological finding	No
8	82	m	29 mm Evolut R PRO	Dislocation into ascending aorta after loss of capture during postdilation due to severe regurgitation	Sapien 3 Ultra 26 mm	4	Descending aorta	No pathological finding	No
9	84	f	Portico 27 mm	“Pop-up” after valve release	Sapien 3 Ultra 23 mm	2	Ascending aorta	Hypoattenuated leaflet thickening at embolized valve	No
10	84	m	29 mm Navitor	“Pop-up” after valve release	Sapien 3 29 mm	2	Ascending aorta	Hypoattenuated leaflet thickening at embolized valve	No

f, female; m, male; TAVI, transcatheter aortic valve implantation; THV, transcatheter heart valve; TVEM, transcatheter valve embolization and migration.

**FIGURE 2:**
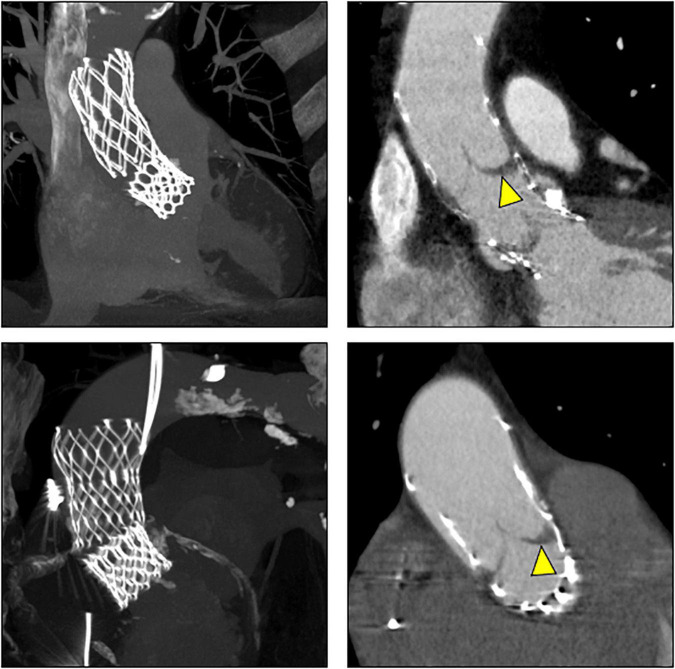
Subclinical valve thrombosis in embolized valves. In patients 9 (top) and 10 (bottom; see Table 5 for details), follow-up CT detected hypoattenuated leaflet thickening (arrow heads) in self-expanding valves embolized into the ascending aorta.

**FIGURE 3:**
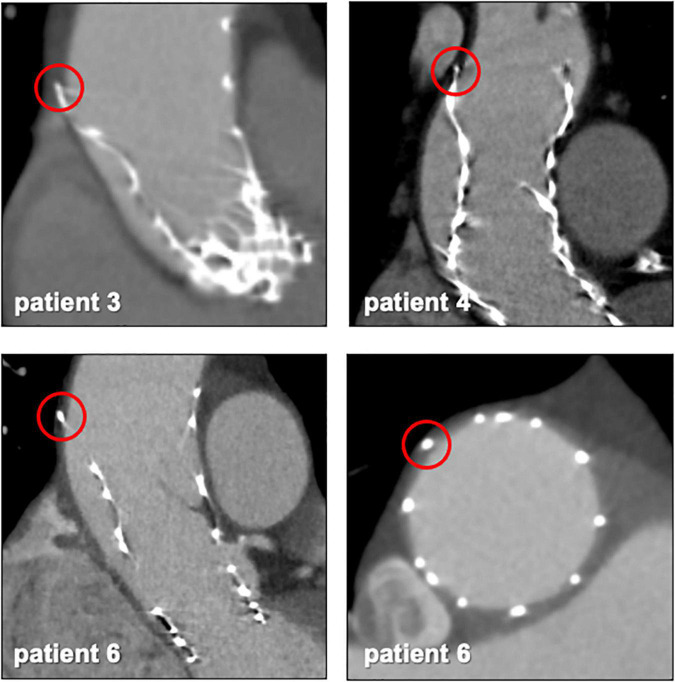
Protruding stent frames into the aortic wall. CT follow-up images from patients 3, 4, and 6 (Table 5) revealed parts of the upper crown of the stent frame protruding into the aortic wall.

CT exams were performed on average 26.3 months after TAVI (range 2–84 months). Similar to the overall distribution in all TVEM patients (Figure 1B), the embolized valve was left in the aortic root or the ascending aorta in eight of the examined patients. Hypoattenuated leaflet thickening (HALT) was detected in two embolized valves (Figure 2). In these two patients stent frames showed no deformation. In addition, parts of the stent frame protruding into the aortic wall, yet without signs of dissection, were observed in three patients (Figure 3).

**FIGURE 4:**
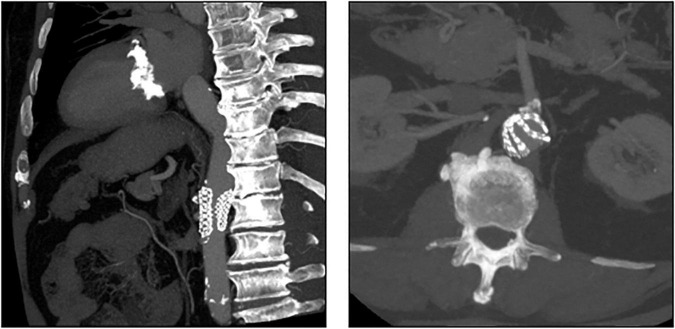
CT images of a patient with embolization of an Evolut PRO. In this case (patient number 8 from Table 5), the snare used to pull the embolized Evolut PRO further into the ascending aorta was entangled in the valve frame. The bent Evolut PRO was eventually pulled into the descending aorta where the snare could be liberated.

**FIGURE 5:**
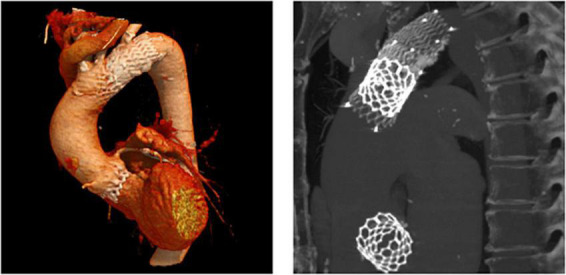
CT images of a patient with embolization of a Sapien 3. CT follow-up of patient number 7 (Table 5). After embolization due to loss of capture during implantation, the embolized Edwards Sapien 3 was pulled back into the proximal aortic arch by the semi-inflated delivery balloon and affixed using two self-expanding stents.

In patient 8, the embolized valve remained in the descending aorta. In this particular case, the snare used to pull the embolized Evolut R further into the ascending aorta (to avoid coronary obstruction) was stuck within the valve frame. The bent Evolut PRO was eventually pulled into the descending aorta where the snare could be liberated (Figure 4). Corresponding to the overall lower incidence of TVEM during TAVI using a balloon-expandable valve, only two patients underwent CT follow-up after embolization of an Edwards Sapien 3 caused by loss of capture during implantation. In patient 1, the embolized valve remained in the aortic root and was secured by valve-in-valve-implantation of a second Sapien. In patient 7, management of the TVEM was complicated by a combination of an aneurysm of the ascending aorta and a narrow, calcified arch. Consequently, the embolized valve could neither be implanted into the wide ascending aorta nor withdrawn into the descending aorta. Instead, it was gently pulled back by the semi-inflated delivery balloon as far as possible into the proximal aortic arch and affixed by two self-expanding stents (Figure 5).

## Discussion

TAVI is a well-established interventional treatment option for aortic stenosis and a large number of studies have evaluated its safety and efficacy compared to surgical valve replacement. However, data on the incidence and long-term consequences of TVEM in TAVI patients is scarce.

In our single-center cohort of 3757 TAVI patients, 54 TVEM occurred over the course of 12 years. The rate of TVEM in our center (1.44%) falls well within the previously published range of 0.3–1.7% (2, 10, 11). Incidence of TVEM in our registry was higher in the beginning of the study period and then decreased over the years (up to 3.4% 2009–2013, 0.55% in 2020). Many potential causes (e.g., growing experience, technical evolution of the valves, release dates of new generations, decreasing morbidity of patients suitable for TAVI, changes in access evaluation/sizing of valve) were previously described in literature (2) and are excellently summarized by Landes et al. (3). Analysis of procedural reports and images identified spontaneous embolization of self-expanding valves into the ascending aorta (“pop-up”), migration into the LV, incomplete release of the valve from the delivery system, embolization during postdilatation and loss of capture during implantation as the main causes of TVEM over the study period (Figure 1A). In accordance with data from a large registry previously published by Kim et al., TVEM could be managed interventionally in the majority of cases but led to conversion to surgery in 14.8% of TVEM patients (2). In 80% of TVEM cases, the embolized valve was left in the ascending aorta (Figure 1B).

In agreement with previous data, comparison of TVEM patients to a contemporary cohort of 200 consecutive patients undergoing TAVI for aortic native valve disease identified the use of a self-expanding valve (OR 4.63, CI 2.2–9.7) and the presence of a horizontal aorta (OR 7.51, CI 3.4–16.6) as the main risk factors for TVEM (12, 13). Recently, the impact of a horizontal aorta on procedural success was examined by Abramovitz et al. (7). In patients who underwent TAVI with a SEV, an inverse relationship between horizontal aorta and acute procedural success was shown. In addition to a more difficult THV positioning, TAVI in horizontal aortas is associated with a higher rate for postdilatation – another major mechanism for TVEM identified in our cohort (Figure 1A). In addition, reduced left ventricular ejection fraction was associated with a higher risk of TVEM (OR 2.94, 95% CI 1.1–7.3, *p* = 0.016). Rapid valve release to shorten low flow periods as well as avoidance of multiple implantation attempts for the optimization of implantation height might contribute to a higher risk for TVEM in HFrEF patients.

Furthermore, it seemed that smaller annular size (22.9 ± 1.6 mm in TVEM vs 24.3 ± 2.4 mm in the total cohort, *p* = 0.187) are more prone to embolization, yet statistical significance was not reached, therefore the probability of chance cannot be excluded.

**FIGURE 6:**
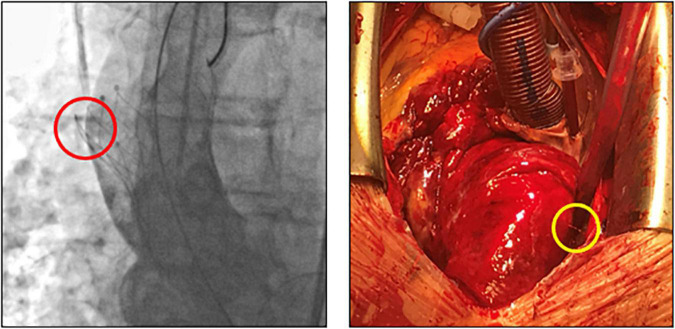
Fluoroscopic images and intraoperative situs after perforation of the ascending aorta by an embolized self-expanding valve. Fluoroscopy (left) and intraoperative situs (right) of a patient with hemorrhagic shock due to perforation (circles) of the ascending aorta. After embolization, the self-expanding valve (25 mm Portico) was deliberately pulled further into the ascending aorta to avoid coronary obstruction.

Subclinical leaflet thrombosis characterized by hypoattenuated leaflet thickening (HALT) is a frequent finding in transcatheter bioprosthetic aortic valves with a prevalence of up to 28% in short- and long-term CT follow-up (4, 5, 14). To our knowledge, our study is the first to systematically examine embolized valves by CT after mid- to long-term follow-up. HALT was detected in the embolized valves in two TVEM patients (Figure 2). Since the number of patients in our imaging sub-study is low it is not possible to draw definite conclusions. Of note, both patients with HALT did not take oral anticoagulants which have been shown to prevent the formation of leaflet thrombosis. In addition, our findings advise some caution as parts of the upper crown of embolized self-expanding valves protruding into the aortic wall were observed in three patients (Figure 3). This is reminiscent of another case from our TVEM cohort complicated by valve embolization due to pop-up of a 25 mm Portico self-expanding valve. The embolized valve was snared and pulled into the ascending aorta to avoid coronary obstruction. After successful implantation of a second transcatheter valve (23 mm Edwards Sapien 3) the patient developed hemorrhagic shock. Angiography revealed perforation of the ascending aorta by a part of the upper crown of the THV protruding through the aortic wall. The valve was surgically removed, and the ascending aorta repaired on cardiopulmonary bypass (Figure 6). Accordingly, interventionalists should be aware of this potential complication when embolized valves have to be actively pulled up into the ascending aorta using a snare. Patients should be examined by CT in a timely fashion to rule out perforation of the ascending aorta if they develop hemodynamic instability in the postinterventional course.

## Limitations

Our results are mainly limited by the retrospective design of our analysis and the low number of patients undergoing follow-up examination by CT. Conclusions in regard of the CT scans should be put in the context of different timing due to the retrospective design. While comparison of age and sex showed that our control sample was representative of our entire TAVI cohort, we cannot exclude the possibility that the prevalence of comorbidities or anatomical features (e.g., aortic angle) changed over time.

Confidence intervals of the odds ratios of our regression model are wide despite the good fit of our model validated by Nagelkerke’s R square. Furthermore, our analysis is limited by a potential survivor bias that might miss relevant long-term complications in TVEM patients. However, data from Kim et al. suggest that the major impact on morbidity and mortality of TVEM is limited to the short-term follow-up period after TVEM (2).

## Conclusion

In this cohort comprising 3757 patients, TVEM occurred in 1.44%. Most cases can be managed interventionally. Predisposing risk factors for TVEM are horizontal aorta, the use of self-expanding valves and HFrEF. In four out of five cases, the embolized valve remains in the ascending aorta. Importantly, follow-up examinations by CT did not detect relevant pathological findings requiring intervention in patients after TVEM. However, the possibility of subclinical leaflet thrombosis and of protrusion of parts of the stent frame in the aortic wall should advise caution.

## Data availability statement

The original contributions presented in this study are included in the article/supplementary material, further inquiries can be directed to the corresponding author.

## Ethics statement

The studies involving human participants were reviewed and approved by Ethikkommission der Charité – Universitätsmedizin Berlin. The patients/participants provided their written informed consent to participate in this study.

## Author contributions

HD and DF designed the study and co-wrote the first draft of the manuscript. DF performed the statistical analysis. MP, AK, and DF recruited patients and acquired and analysed the data. AB and FKn performed and analyzed the echocardiographic examinations. HD, KS, ML, DL, UL, FKr, MS, SS, and HG analyzed procedural reports and angiograms and co-wrote the manuscript. AL and SN performed and analyzed the CT angiograms. All authors contributed to the article and approved the submitted version.

## Conflict of interest

HD, KS, ML, DL, UL, and MS were received financial research support and speakers’ fees from Abbott, Edwards LifeSciences and Medtronic. The remaining authors declare that the research was conducted in the absence of any commercial or financial relationships that could be construed as a potential conflict of interest.

## Publisher’s note

All claims expressed in this article are solely those of the authors and do not necessarily represent those of their affiliated organizations, or those of the publisher, the editors and the reviewers. Any product that may be evaluated in this article, or claim that may be made by its manufacturer, is not guaranteed or endorsed by the publisher.

## Abbreviations

TVEM: trancatheter valve embolization and migration
TAVI: transcatheter aortic valve implantation
ECG: electrocardiogram
CT: computed tomography
VARC 2: Valve Academic Research Consortium – 2
TTE: transthoracic echocardiography
EACVI: European Association of Cardiovascular Imaging
SEV: self-expanding valve
BEV: balloon-expandable valve
HFrEF: heart failure with reduced ejection fraction
BMI: body mass index
HALT: hypoattenuated leaflet thickening.
